# Evaluation of the real-time fluorescence loop-mediated isothermal amplification assay for the detection of *Ureaplasma urealyticum*

**DOI:** 10.1186/s13568-022-01357-2

**Published:** 2022-02-11

**Authors:** Jie-Ni Shen, Jing-Yi Ye, Meng-Xiao Lao, Chu-Qiao Wang, Dong-Hong Wu, Xiao-Ying Chen, Li-Hong Lin, Wen-Yan Geng, Xu-Guang Guo

**Affiliations:** 1 Department of Clinical Laboratory Medicine, The Third Affiliated Hospital of Guangzhou Medical University, Guangzhou, China; 2 Department of Clinical Medicine, The Nanshan School of Guangzhou Medical University, Guangzhou, China; 3 Department of Clinical Laboratory Medicine, Maternal and Child Health Care Hospital of Guangming District, Shenzhen, China; 4 Center for Reproduction Medicine, Key Laboratory for Major Obstetric Diseases of Guangdong Province, The Third Clinical School of Guangzhou Medical University, Guangzhou, China; 5 Department of Blood Transfusion, The Third Affiliated Hospital of Guangzhou Medical University, Guangzhou, China; 6 Key Laboratory for Major Obstetric Diseases of Guangdong Province, The Third Affiliated Hospital of Guangzhou Medical University, Guangzhou, China; 7 Key Laboratory of Reproduction and Genetics of Guangdong Higher Education Institutes, The Third Affiliated Hospital of Guangzhou Medical University, Guangzhou, China

**Keywords:** *Ureaplasma urealyticum*, Loop-mediated isothermal amplification, Real-time

## Abstract

*Ureaplasma urealyticum* (UU) is commonly present in human reproductive tract, which frequently leads to genital tract infection. Hence, there is an urgent need to develop a rapid detection method for UU. In our study, a real-time fluorescence loop-mediated isothermal amplification (LAMP) assay was developed and evaluated for the detection of UU. Two primers were specifically designed based on the highly conserved regions of *ureaseB* genes. The reaction was carried out for 60 min in a constant temperature system using *Bst* DNA polymerase, and the process was monitored by real-time fluorescence signal, while polymerase chain reaction (PCR) was performed simultaneously. In real-time fluorescence LAMP reaction system, positive result was only obtained for UU among 9 bacterial strains, with detection sensitivity of 42 pg/μL (4.2 × 10^5^ CFU/mL), and all 16 clinical samples of UU could be detected. In conclusion, real-time fluorescence LAMP is a simple, sensitive, specific and effective method compared with conventional PCR, which shows great promise in the rapid detection of UU.

## Introduction

Reproductive tract infection caused by *Mycoplasma hominis* (MH), *Mycoplasma genitalium,* and *Ureaplasma urealyticum* (UU), imposes a threat to human reproductive tract health. Among these, UU is the most prevalent pathogen that colonizes the human reproductive tract (Esen et al. 2017). Additionally, UU causes severe problems for infants and their mothers. As an example, research has proved that UU could be vertically transmitted from mother to child, thereby increasing morbidity and mortality of newborns (Kafetzis et al. 2004; Sobouti et al. 2014). Moreover, people who have a mixed infection of UU and MH are prone to cause premature delivery (Kwak et al. 2014; Lee et al. 2016).

Conventional diagnosis of UU infection relies on positive culture, antigen detection, serology, and molecular biology. Generally, polymerase chain reaction (PCR) is one of the most common techniques (Liu et al. 2017). However, the above-mentioned methods require either long culture periods or precision instruments, thus limiting their application in primary medical institutions, especially in developing countries. Apart from that, PCR increases the risk of sample cross-contamination (Dehghan Esmatabadi et al. 2015). Hence, an alternative diagnostic is needed to diagnose UU infection fast and efficiently.

Loop-mediated isothermal amplification (LAMP), which was first described by Notomi in 2000 (2000), is a promising alternative to conventional PCR techniques. It requires at least 4 specific primers to identify 4–6 distinct sequences of DNA template, which can be less influenced by inhibitory molecules in clinical samples (Enomoto et al. 2005). Due to the characteristics of primers, the concentration of LAMP is higher than that in PCR reaction. The temperature of real-time fluorescence LAMP reaction ranged between 60 and 65 ℃, and it merely takes *Bst* DNA polymerase 30–60 min to amplify target DNA sequence (Ruang-Areerate et al. 2021). Compared with conventional PCR, real-time LAMP increases the reaction rate two to three times (Guo et al. 2018).

The LAMP-amplified products can be observed directly by reaction tubes, and thus avoiding the requirement of thermal cycling and electrophoresis, which enable bedside of *Ureaplasma* spp. (Fuwa et al. 2018). Hence, with addition of fluorescent dye, real-time fluorescence LAMP shows tremendous ability of quick quantitative detection of UU infection. Though LAMP technology has been utilized for UU detection in clinical research (Fuwa et al. 2018), no research has focused on the establishment and evaluation of UU detection based on real-time fluorescence LAMP. Our study aims to evaluate real-time fluorescence LAMP assay with a modification, which combined with high sensitivity, specificity and efficiency, to investigate its feasibility in clinical practice and facilitate the rapid detection of UU in clinical samples.

## Methods

The study was approved by the institutional ethics committee of Third Affiliated Hospital of Guangzhou Medical University. All patients provided written consent before sample collection.

### Bacterial strains and bacterial genomic DNA extraction

The strains were collected from the Department of microbiology laboratory, the Third Affiliated Hospital of Guangzhou Medical University, where the strains were isolated, identified by VITEK 2 automatic microorganism identification instrument, and stored at − 70 °C. The sources of these genomes are shown in Table 1. The strains used in the present study include *Ureaplasma urealyticum* ATCC27618, *Escherichia coli, Proteus mirabilis, Klebsiella pneumoniae, Enterococcus aerogenes, Staphylococcus haemolyticus, Pseudomonas aeruginosa, Streptococcus agalactiae and Mycoplasma hominis*. The protocol was performed as follows. Bacteria suspensions (1 mL) were prepared from the bacteria colonies in a liquid medium washed by phosphate buffer saline. The extraction of bacteria DNA was conducted under instruction manuals. Finally, concentration and purity of the extracted specimens were tested on a DNA concentration tester.

**Table 1:** Bacterial strains used in this study

*Ureaplasma urealyticum* ATCC27618	
*Escherichia coli*	
*Proteus mirabilis*	
*Klebsiella pneumoniae*	
*Enterococcus aerogenes*	
*Staphylococcus haemolyticus*	
*Pseudomonas aeruginosa*	
*Streptococcus agalactiae*	
*Mycoplasma hominis*	

The source of all bacterial strains was: Third Affiliated Hospital of Guangzhou Medical University

### Primer design and real-LAMP system

The primers were designed by the real-time fluorescence LAMP primer design software Primer Explorer V4 based on DNA sequence of UU *ureaseB* gene (GenBank accession no. AF085726.2). The sequences of primers are listed in Table 2. Primers included forward outer primer (F3), backward outer primer (B3), forward internal primer (FIP), backward internal primer (BIP), loop forward primer (LF), and loop backward primer (LB), which were synthesized by Invitrogen (Shanghai, China). Bacterial genomic DNA extraction kit was purchased from Tiangen Biochemical Technology Company Limited.

**Table 2:** Primers used for the real-time fluorescence LAMP and PCR reaction system

UreaseB-1 Primers	Sequence (5ʹ–3ʹ)	Length (bp)
F3	AGGAGATAATGATTATATGTCAGGA	25
B3	TAACGCTATCACCAGTTGTG	20
FIP(F1c + F2)	CAACTTGGATAGGACGGTCACCAATTAGTACCAGGAGCAATTAACT	46
BIP(B1c + B2)	ATTCCATCAGGTACTGCTATTCGTTTTCCGTTAACTAAGCCGTT	44
LOOPF	TCTCTACCTTCGTTCATCACAATT	24
LOOPB	TTAGTCGGAACACGTGAAGTT	21

UreaseB-2 Primers	Sequence (5ʹ–3ʹ)	Length (bp)
F3	AGGAGATAATGATTATATGTCAGGA	25
B3	CACCAGTTGTGATACCATATAGAT	24
FIP(F1c + F2)	CAACTTGGATAGGACGGTCACCAATTAGTACCAGGAGCAATTAACT	46
BIP(B1c + B2)	ATTCCATCAGGTACTGCTATTCGTTTTCCGTTAACTAAGCCGTT	44
LOOPF	TCTCTACCTTCGTTCATCACAATT	24
LOOPB	TTAGTCGGAACACGTGAAGTT	21

UU PCR primer	Sequence (5ʹ–3ʹ)	Length (bp)
UU forward	CAGGATCATCAAATCAATTCAC	22
UU reverse	CATAATGTTCCCCTTCGTCTA	21

The LAMP reaction system was established according to the manual of DNA amplification kit. The LAMP mix without DNA templates was first prepared on ice (Table 3). Two microliters of DNA template were added to 23 µL of LAMP mix, to a total volume of 25 µL. Sterile distilled water was used as a negative control in all tests. The fluorescence channel was selected for TIANGEN kits (Tiangen; Beijing, China) detection. The mix reaction was incubated in a real-time quantitative PCR analyzer (ABI 7500) at 63 °C for 60 min, then 80 °C for 2 min to terminate the reaction. Fluorescent dye of SYTO-9 (Guangzhou Deaou Biotechnology) was added to the tubes. The reaction was considered positive if green fluorescence was observed.

**Table 3:** Composition of the real-time fluorescence LAMP reaction mix

	Components	Volume
Reaction mix	20 mM Tris–HCl (pH 8.8)	12.5 μL
	10 mM KCl	
	8 mM MgSO_4_	
	10 mM (NH_4_)_2_SO_4_	
	0.1% Tween-20	
	1 mM Betaine	
	1.6 mM dNTP	
Primer mix	FIP (1.6 μM) and BIP (1.6 μM)	1 μL
	F3 (0.2 μM) and B3 (0.2 μM)	
	FLF (0.8 μM) and FLB (0.8 μM)	
Nuclease-free water		8 μL
Bst DNA polymerase		1 μL
SYTO-9		0.5 μL
DNA template		2 μL
Total volume		25 μL

Reaction condition: 63 °C, 45–60 min

### Primer screening experiment and dissociation curve analysis

Two selected primers, *UreaseB-1* and *UreaseB-2*, were prepared for working solutions. The sampling along with the startup of reaction procedure was executed in line with instructions of DNA amplification kit. Meanwhile, negative controls were also performed for each reaction tube. The working solutions were prepared using selected primers. A reaction tube was used for dissolution curve detection and to compare the amplification efficiency of two sets of primers while screening out the highest amplification efficiency without a primer dimer.

### Specificity test of real-time fluorescence LAMP

The genomic DNA of UU standard strain (ATCC 27618) and other common clinical pathogens (including *Proteus mirabilis, Klebsiella pneumoniae, Enterococcus aerogenes, Staphylococcus haemolyticus, Pseudomonas aeruginosa, Streptococcus agalactiae, Mycoplasma hominis, Escherichia coli*) were extracted and amplified in identical reaction conditions of real-time fluorescence LAMP. The specificity of primers was evaluated.

### Sensitivity test of real-time fluorescence LAMP

The concentration of DNA was quantified with the help of a Thermo Scientific Nanodrop 2000 spectrophotometer after titering with 1 μL of TE buffer. The sensitivity of the reaction was assessed using a serial dilution of genomic DNA template, wherein original 2 μL of genomic DNA was diluted tenfold with ultra-pure water five times. Six concentration gradients were finally attained, which were marked B1, B2, B3, B4, B5, B6 in order. The real-time fluorescence LAMP reactions with identical total volume and different DNA templates with six concentrations were performed under identical conditions, and the sensitivity of detection was assessed according to amplification curves.

### Repeatability test of real-time fluorescence LAMP

The genomic DNA from one positive bacterial strain and one negative bacterial strain was used for repeatability assessment of real-time fluorescence LAMP reaction. The LAMP reactions were performed three times under the same experimental conditions. The same primer used for a repeat test was used to evaluate the reliability of LAMP.

### PCR analysis

PCR detection was amplified in a 25 μL volume consisting of 5 μL template, 0.5 U of TaKaRa Ex Taq, 0.2 mmol/L deoxynucleotide mixture, and 10 × TaKaRa Ex Taq Buffer. For the UU assay, 0.8 μmol/L each of the primers UU forward and UU reverse was used in Table 1 (Kong et al. 2000). Amplification conditions were set with an initial denaturation step for 60 s followed by 30 cycles for denaturation at 94 °C for 30 s, primer annealing at 55 °C for 30 s, and extension at 72 °C for 60 s with a final incubation at 72 °C for 15 min. Thermal profiles were performed on a C1000™ Thermal Cycler (Bio-Rad, Hercules, CA, USA). The Amplified products were analyzed by a microchip electrophoresis system (Bio-Rad, Hercules, CA, USA).

### Detection of clinical samples

Clinical strains were collected from clinical patients who tested positive for UU after solid culture. The secretions were inoculated in UU liquid medium and cultured at 37 ℃ for 36–48 h. UU growth when the color of the culture tube changed from yellow to pink, and the liquid clarified. Then the culture medium was filtered and transferred to a solid culture medium for confirmation. UU was positive with the tiny colonies with brown, characteristic 15–50 μm, under a phase-contrast microscope. Then, the extracted DNA samples isolated from 16 clinical specimens were used as the templates for PCR and LAMP assays.

### Statistical analysis

The counting data were analyzed by Kappa test performed with SPSS 19.0 software (IBM Corporation, Armonk, NY, USA) at 95% confidence intervals (CI). The clinical sensitivity, specificity, positive predictive values (PPV), and negative predictive values (NPV) of real-time fluorescence LAMP and PCR assays were evaluated using conventional culture as a gold standard method.

## Results

### Primer screening test

**Fig. 1:**
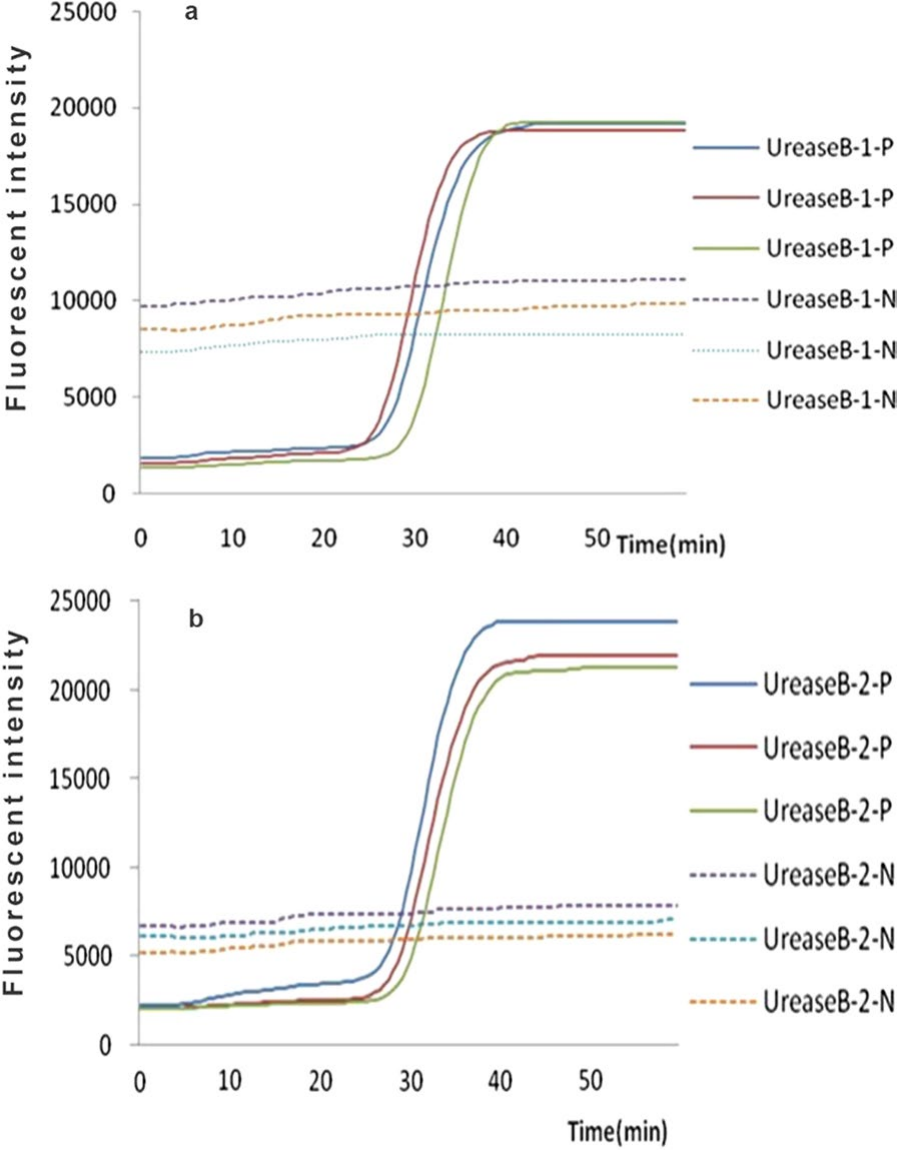
The primers of real-time fluorescence LAMP screening test

Two sets of amplified primers with no-loop structure products showed each reaction curve (Fig. 1). The visually detectable reaction peak emerged earlier in the reaction with primer *UreaseB-2* as compared with primer *UreaseB-1*, and the fluorescent intensity was also higher in the reaction with primer *UreaseB-2* than with primer *UreaseB-1* at each check-point. The *UreaseB-2* was the most efficient primer, which had a peak at 40 min after the initiation of reaction. Also, no amplification was observed in the negative control. Therefore, primer *UreaseB-2* had relatively higher amplification efficiency for UU *ureaseB* gene and was selected for subsequent experiment.

### Specificity of real-time fluorescence LAMP

**Fig. 2:**
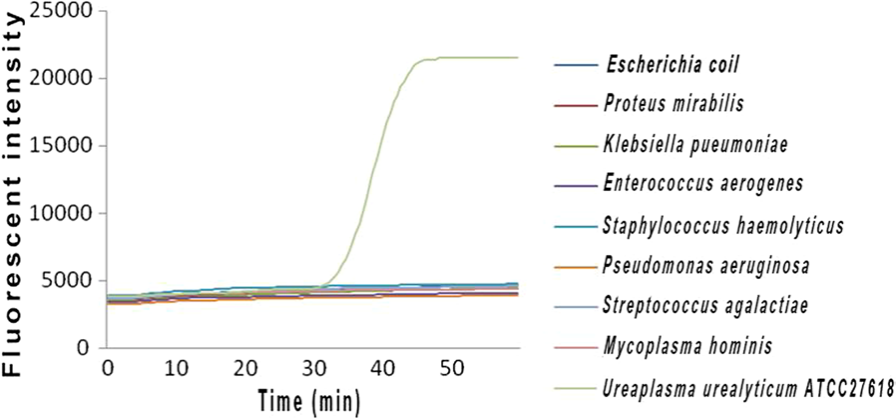
The specificity of real-time fluorescence LAMP

In the present study, eight common pathogenic bacteria were selected as negative controls and no amplification was tested by real-time fluorescence LAMP assay (Fig. 2). Therefore, the primers for real-time fluorescence LAMP had high specificity, without cross-reaction or false-positive results.

### Sensitivity of real-time fluorescence LAMP

**Fig. 3:**
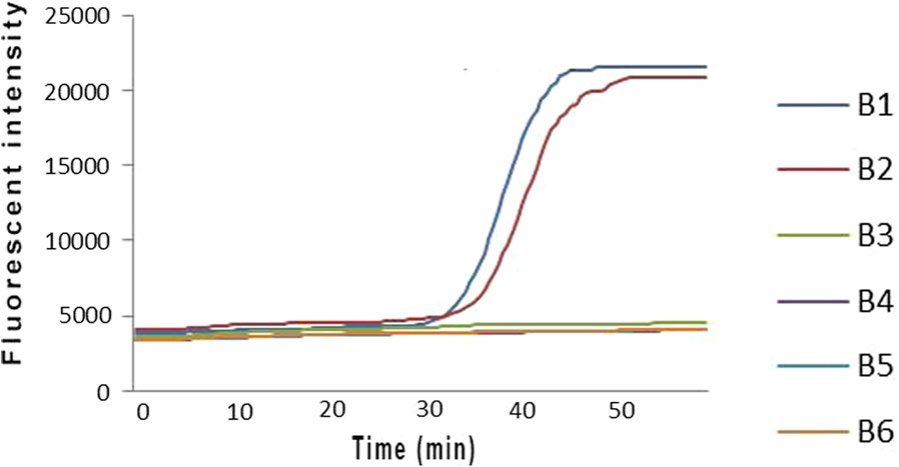
The sensitivity of real-time fluorescence LAMP

The concentration of UU genomic DNA was 420 pg/μL, which was diluted by five gradients. Therefore, the resulting concentrations corresponded to 42, 4.2, 0.42, 0.042, 0.0042 pg/μL. The sensitivity of UU was 42 pg/μL (Fig. 3). These concentrations correspond to the bacterial concentrations 4.2 × 10^6^, 4.2 × 10^5^, 4.2 × 10^4^, 4.2 × 10^3^, 4.2 × 10^2^ and 4.2 × 10^1^ CFU/mL, respectively. No double-peak appeared on the melting curve with the DNA template at six concentrations, indicating that there was no formation of primer dimers.

### Repeatability of real-time fluorescence LAMP

**Fig. 4:**
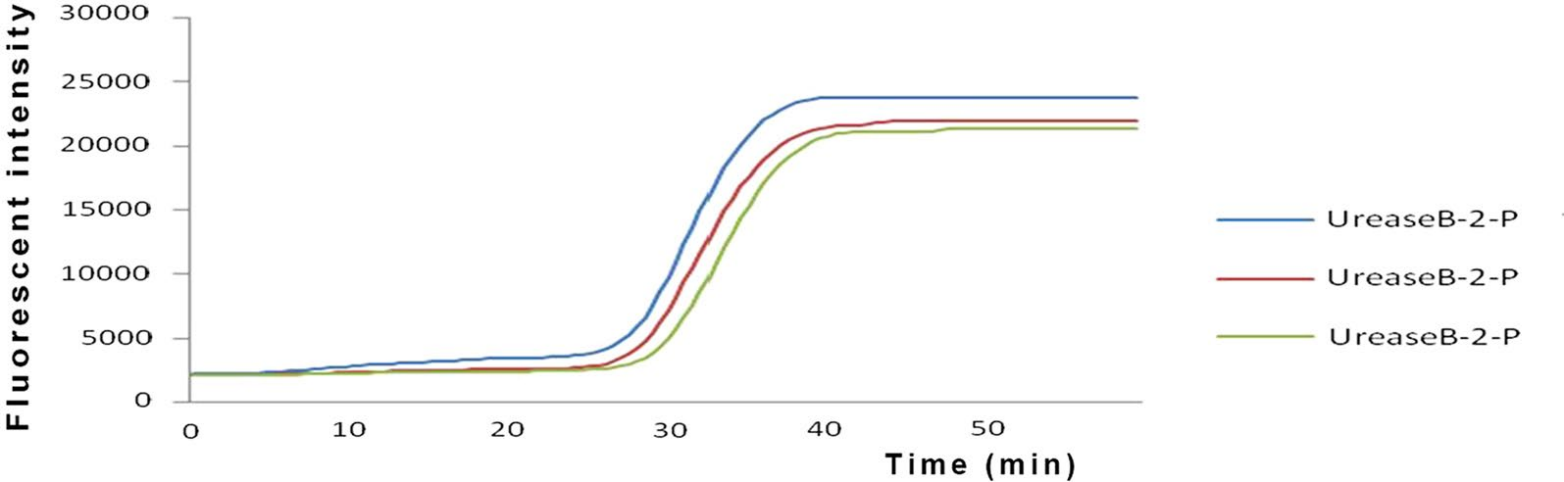
The repeatability of real-time fluorescence LAMP

The reaction peak was visually detected almost simultaneously for the triplicate tubes of a positive sample, and the amplification curves nearly overlapped (Fig. 4). The results showed that real-time fluorescence LAMP assay had satisfactory repeatability.

### Detection of clinical samples

**Fig. 5:**
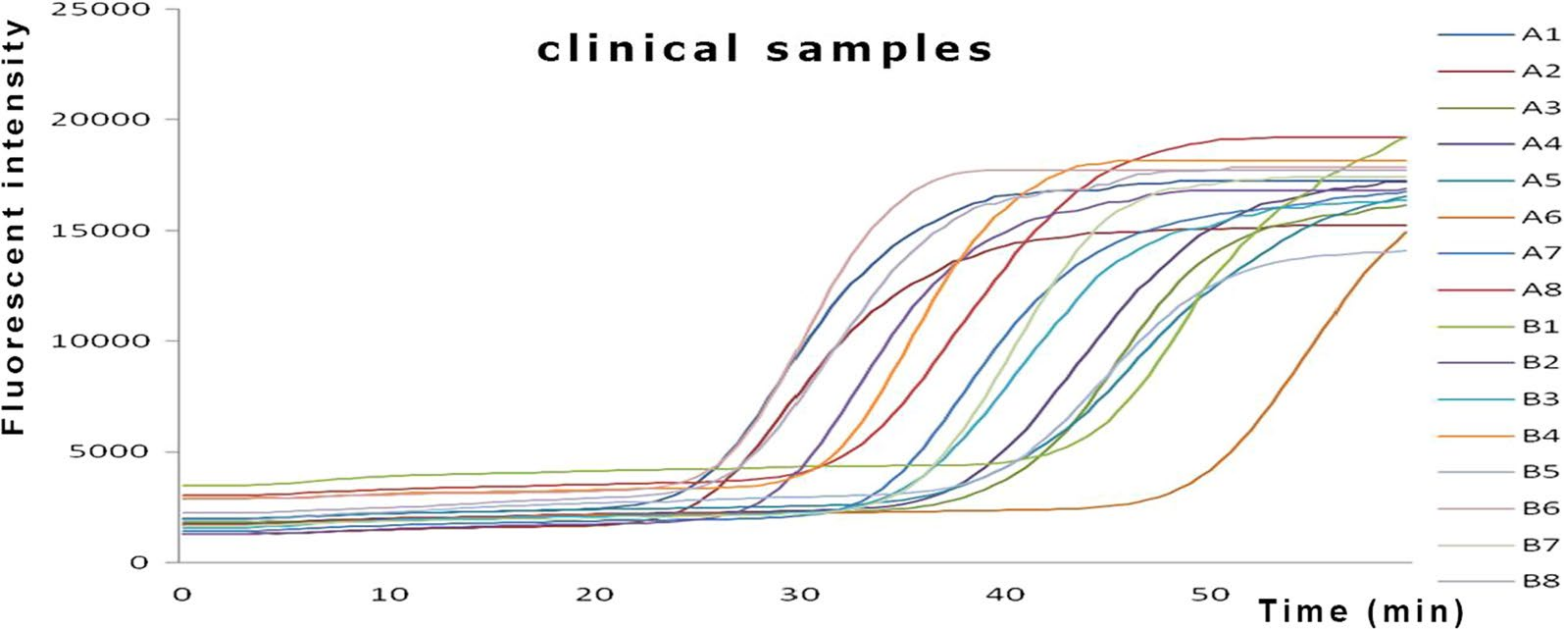
Detection of common clinical samples by real-time fluorescence LAMP

Real-time fluorescence LAMP method established in this study and conventional PCR method were used to detect UU in 16 clinical samples simultaneously. Results (Fig. 5) showed that 16 clinical samples were detected positive by LAMP method, while only 14 samples were confirmed to be positive by PCR, showing real-time fluorescence LAMP is superior to PCR in sensitivity.

The overall performances of real-time fluorescence LAMP, conventional PCR, and culture tests used for the detection of UU are shown in Table 4. The sensitivity and PPV of LAMP were 100% (16/16 specimens; 95% CI 79.4–100%) and 100% (16/16; 95% CI 79.4–100%), respectively, compared to culture results. In contrast, the results of conventional PCR were 87.5% (14/16; 95% CI 61.7–98.5%) and 100% (14/14; 95% CI 76.8–100.0%), respectively.

**Table 4:** Comparison of real-time fluorescence LAMP, PCR, and culture results from 16 clinical specimens

Detection of UU		Culture^a^			Sensitivity (95% CI)	Specificity (95% CI)	PPV (95% CI)	NPV (95% CI)
		Pos	Neg	Total				
Real-time fluorescence LAMP^b^	Pos	16	0	16	100% (79.4–100)16/16	NA	100% (79.4–100)16/16	NA
	Neg	0	0	0				
PCR^c^	Pos	14	0	14	87.5% (61.7–98.5)14/16	NA	100% (76.8–100)14/14	NA
	Neg	2	0	2				
	Total	16	0	16				

*PPV* positive predictive value, *NPV* negative predictive value

^a^Culture was performed on UU medium. Pos indicates that a UU strainwas isolated, and Neg indicates that no UU strain was isolated

^b^Real-time fluorescence LAMP reaction was confirmed using a real-time quantitative PCR analyzer (ABI 7500). Pos indicates that amplification occurred in the UU LAMP assay, and Neg indicates that amplification did not occur in the UU LAMP assay

^c^PCR was confirmed using an electrophoretic analysis (C1000™ Thermal Cycler). Pos indicates that amplification occurred with the UU PCR assay, and Neg indicates that amplification did not occur in the UU PCR assay

## Discussion

UU is a common human genital tract colonizer, which is frequently related to premature delivery, cesarean section, placental inflammation, fetal lung damage and infant death (Bayraktar et al. 2010). Therefore, rapid detection of UU is of great importance for early diagnose of UU infection.

Conventional diagnosis of UU is based on liquid culture and PCR. Culture is still a gold standard for the detection of UU and provides additional data on antimicrobial sensitivities. However, besides time consumption, conventional culture cannot accurately determine the presence or absence of UU infection, showing there is a certain false-positive rate (Gerber et al. 2003). PCR has been reported to be more sensitive, specific, and rapid than conventional culture method. The clinical sensitivity and specificity of conventional PCR methods have been reported to be from 94 to 95% and from 91 to 98%, respectively (Abele-Horn et al. 1996; Povlsen et al. 1998). However, PCR-based techniques relied on sophisticated apparatus and complex sample-handling procedures, which limited their application in primary medical facilities. Therefore, given the drawbacks and limitations of conventional detection technologies, an efficient and low-cost method of detecting UU was established in this study based on a real-time fluorescence LAMP assay.

Real-time fluorescence LAMP is a combination of real-time fluorescent technology and isothermal amplification technology with the advantages of being simple, rapid and highly sensitive. LAMP uses *Bst* DNA polymerase and 4–6 primers that are specific to 4–6 separate regions of target sequences, wherein the reaction is performed in a homothermal condition (Parida et al. 2004). Initially, four primers are used altogether but later, only the inner primers are needed in the cycling reaction. In LAMP reaction, the inner primer hybridizes to target DNA region which gives rise to the formation of a stem-loop DNA pattern, and the LAMP cycling reaction starts (Kuboki et al. 2003; Yang et al. 2011). Amplification product shows a characteristic ladder band, and the process is related to the formation of magnesium pyrophosphate (Mori and Notomi 2009). Therefore, LAMP assay allows visualization by naked eye, eliminating the need for cumbersome electrophoresis and ultraviolet observation (Xia et al. 2014), which shows its rapid detection of pathogenic microorganisms. In particular, without the necessity of using expensive thermal circulators, gel electrophoresis and UV detection equipment, LAMP is low-cost. Currently, quantitative LAMP has been developed in broad spectrums including cheap semi-quantified *E. coli* LAMP using paper-based on costly LAMP chips (Rolando et al. 2019; Saengsawang et al. 2021). It is suitable for the rapid detection of a pathogenic microorganism on the spot, the popularization of field and grass-roots in wartime (Mori et al. 2013).

The readout of conventional LAMP mainly relies on white precipitates. However, weak positive results were obtained due to poor amplification efficiency or minute amounts of amplified products (Guo et al. 2018). To overcome this drawback, the fluorescent dye SYTO-9 was used in the LAMP reaction and ABI 7500 system was employed. A real-time fluorescent quantitative PCR instrument was utilized as the LAMP platform, which allowed dynamic and quantitative monitor with increased detection sensitivity. However, SYTO-9 could bind to double-stranded DNA if there was a dimer formation of primers, which could easily lead to false-positive results. To detect potential false-positive results, the melting curve was analyzed and no double-peak was found, indicating no primer dimer formation during the reaction and high specificity of our assay setup. Furthermore, SYTO-9 was added before the reaction and the whole process was completed with closed lid, which can effectively avoid aerosol pollution caused by open-cover detection (Sukphattanaudomchoke et al. 2020).

In the present study, real-time fluorescence LAMP was performed to detect DNA samples for UU standard strain (ATCC 27618) and 8 other strains of common pathogenic bacteria. Positive amplification was only observed in UU, but not in other strains, showing high specificity of LAMP primers and no cross-reactivity with other non-targeted strains. Using conventional culture as a standard, and the sensitivity of the real-time fluorescence LAMP assay reached 100%, respectively. The high sensitivity of real-time fluorescence LAMP is more impressive when testing with UU standard strain, detecting as low as 42 pg/μL of the DNA concentration, the equivalent of 4.2 × 10^5^ CFU/mL. This sensitivity was further confirmed with 16 parallel samples of the standard strain. Real-time fluorescence LAMP is carried out to detect UU in 16 clinical samples, while PCR is performed simultaneously. Of all clinical samples tested by PCR, 14 samples were confirmed to be positive while real-time fluorescence LAMP had no missed detection.

However, there are still several limitations in our study. First, given that the number of clinical samples is small in our study, larger and more diverse samples could better determine the sensitivity and reliability of the method. Furthermore, LAMP products are not suitable for expression in cloning and other molecular biology due to various stem loop structures (Mansour et al. 2015). In addition, the specificity of real-time fluorescence LAMP could not be compared with a gold standard test because the specimens used for clinical samples examination were culture-positive.

In conclusion, compared with conventional PCR, real-time fluorescence LAMP for UU detection showed advantages of rapid reaction, high sensitivity, specificity, good repeatability and simple operation, which shows promise of bedside tests and application in primary hospitals.

## Data Availability

All data generated or analyzed during this study are included in the article.

## Abbreviations

LAMP: Loop-mediated isothermal amplification
UU: *Ureaplasma urealyticum*
